# New Molecular Reporters for Rapid Protein Folding Assays

**DOI:** 10.1371/journal.pone.0002387

**Published:** 2008-06-11

**Authors:** Stéphanie Cabantous, Yvonne Rogers, Thomas C. Terwilliger, Geoffrey S. Waldo

**Affiliations:** 1 Bioscience Division, Los Alamos National Laboratory, Los Alamos, New Mexico, United States of America; 2 Institut de Pharmacologie et de Biologie Structurale, Centre National de la Recherche Scientifique, Toulouse, France; National Institute on Aging, United States of America

## Abstract

The GFP folding reporter assay [1] uses a C-terminal GFP fusion to report on the folding success of upstream fused polypeptides. The GFP folding assay is widely-used for screening protein variants with improved folding and solubility [2]–[8], but truncation artifacts may arise during evolution, i.e. from *de novo* internal ribosome entry sites [9]. One way to reduce such artifacts would be to insert target genes within the scaffolding of GFP circular permuted variants. Circular permutants of fluorescent proteins often misfold and are non-fluorescent, and do not readily tolerate fused polypeptides within the fluorescent protein scaffolding [10]–[12]. To overcome these limitations, and to increase the dynamic range for reporting on protein misfolding, we have created eight GFP insertion reporters with different sensitivities to protein misfolding using chimeras of two previously described GFP variants, the GFP folding reporter [1] and the robustly-folding “superfolder” GFP [13]. We applied this technology to engineer soluble variants of Rv0113, a protein from *Mycobacterium tuberculosis* initially expressed as inclusion bodies in *Escherichia coli*. Using GFP insertion reporters with increasing stringency for each cycle of mutagenesis and selection led to a variant that produced large amounts of soluble protein at 37°C in *Escherichia coli*. The new reporter constructs discriminate against truncation artifacts previously isolated during directed evolution of Rv0113 using the original C-terminal GFP folding reporter. Using GFP insertion reporters with variable stringency should prove useful for engineering protein variants with improved folding and solubility, while reducing the number of artifacts arising from internal cryptic ribosome initiation sites.

## Introduction

Classical approaches to improve protein solubility include testing various expression conditions [14], varying promoter strength [15], and fusion to various solubility enhancing partners [16]–[18]. However, these strategies do not improve the intrinsic stability and folding success of recalcitrant proteins. In contrast, directed protein evolution can improve the long-term protein stability and folding yield without affecting global protein structure and activity [19]. In this process, mutagenesis is performed randomly on a protein coding sequence and beneficial mutations are selected or screened from the pool of protein variants. Widely-used genetic screens for protein folding and stability include phage display [20], ribosome display and mRNA display [21]. These methods may be followed by additional *in vitro* screens for protein stability and aggregation. These include resistance to proteolysis [22], accessibility of an affinity tag [23], or screens for reduced hydrophobicity [24]. More recently, an elegant method described the use of the twin-arginine transporter (TAT) to screen for correctly-folded protein variants. Folded proteins bearing a fused beta-lactamase are exported into the periplasmic space, conferring resistance to ampicillin [25]. In this approach, proteins are “sandwiched” between an N-terminal TAT transporter tag and C-terminal selectable marker, helping to ensure that selected variants are full-length. However the maximum size of the complexes, and the type of proteins that can be exported by this pathway without bias, remains to be determined.

**Figure 1:**
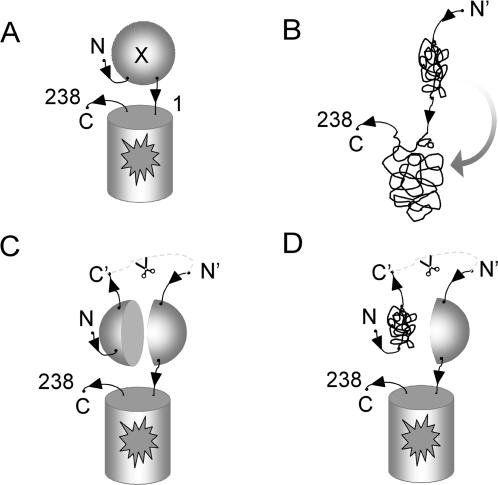
Mechanism proposed for the GFP folding interference assay using C-terminal GFP-based folding reporters. (A) Fluorescence of GFP reflects correct folding of the test protein attached to the reporter. (B) Misfolded fusion proteins interfere with the folding of the downstream GFP domain rendering it non-fluorescent. (C) Internal translation sites or proteolysis can give rise to soluble, truncated polypeptides with new N-termini (*N′, after scissors*) that do not interfere with GFP folding. (D) Truncation artifacts not fused to the GFP escape detection.

**Figure 2:**
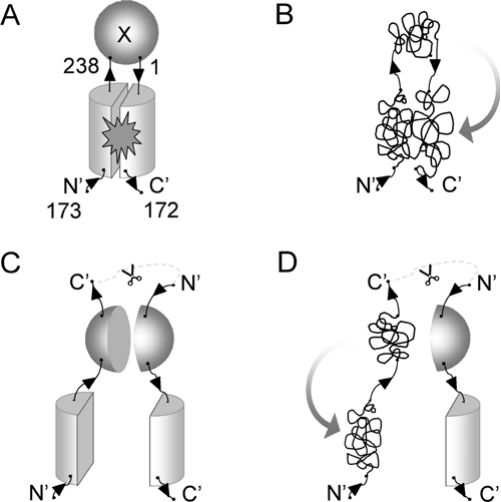
Circular permutant GFP insertion topology reporters. (A) the protein of interest X is inserted between the native N and C termini of GFP. Translation begins at an internal site in the GFP scaffolding (N′), so that the back of GFP is translated first. Correctly folded test inserts do not interfere with the folding and assembly of the GFP fragments. (B) Misfolded inserted target proteins prevent correct reconstitution of the two GFP fragments. (C) Truncations and new internal translation sites produce two independent fragments of GFP, reducing the amount of folded GFP. (D) Misfolded test protein fragments can sequester the fused GFP domain in aggregates preventing GFP assembly.

An alternative for selecting folded proteins consists of fusions with so-called ‘folding reporters’. One example is the use of chloramphenicol acetyl transferase (CAT) to monitor protein folding based on chloramphenicol resistance [26]. Earlier we described a directed evolution approach that combines DNA shuffling mutagenesis followed by selection of variants with improved folding (optima) using the green fluorescent protein (GFP) folding reporter [1]. This approach has been used with large protein complexes, including the 24-subunit ferritin (ca. 450 kDal) [1]. The selection is based on the observation that the fluorescence of GFP fusions is positively correlated with folding of the target protein expressed alone [1]. One explanation of this observation is that poorly folded fusions trap non-productive folding intermediates of the fused GFP domain resulting in so-called ‘folding interference (Fig. 1A, B). Directed protein evolution using cycles of mutagenesis and selection via the GFP folding interference assay has been used to engineer soluble variants of recalcitrant proteins from several organisms [5], [7], [27] including the hexameric NDP kinase from *Pyrobaculum aerophilum* [4]. The GFP folding interference method has also proved useful for finding mutations that reduce the aggregation of the Alzheimer Aβ42 peptide [3], [6], and for identifying chemicals that suppress aggregation of the Alzheimer Aβ42 peptide [28]. Despite these successes, fluorescent ‘false-positive’ truncation artifacts from cryptic internal ribosome binding sites in full-length cDNA coding sequences may arise during directed evolution using C-terminal folding reporters [9]. A small, soluble, truncated polypeptide may be linked to full length fluorescent GFP whether both test protein fragments are soluble (Fig. 1C) or not (Fig. 1D). To help discriminate against these artifacts, we engineered a so-called “circular permutant insertion” GFP reporter, with the test protein inserted between the native N- and C-termini of a GFP circular permutant (Fig. 2A). Misfolding by test proteins would be expected to interfere with the folding and assembly of the two GFP domains (Fig. 2B).We hypothesized that truncation artifacts or translation products from internal ribosome binding sites should be reduced or eliminated when using GFP insertions, since the resulting separated halves of the GFP scaffolding are less likely to associate to form the fluorescent GFP (Fig. 2C), especially when at least one of the fragments is misfolded (Fig. 2D).

Since GFP circular permutants are more likely to misfold [10]–[12] than the conventional topology, we reasoned that circular permutant insertion GFP reporters should exhibit increased sensitivity to target fusion protein misfolding relative to the conventional C-terminal GFP reporter [1]. Moreover, the likelihood of folding interference should decrease as the folding robustness of the GFP domain increases. We recently described a ‘superfolder’ GFP that exhibits reduced folding interference from upstream fused polypeptides compared to folding reporter GFP [13]. GFP hybrid insertion reporters combining mutations from folding reporter GFP [1] and ‘superfolder’ GFP [13] should therefore be tunable to a desired stringency or sensitivity to test protein misfolding. We show here that a set of several hybrid GFP insertion reporters is indeed more sensitive to test protein folding interference and provides a wider dynamic range of sensitivity to test protein misfolding than the C-terminal folding reporter GFP. We use this new insertion topology to discriminate against truncation artifacts that appeared when using the C-terminal GFP folding reporter during directed evolution of an Rv0113 protein variant from *Mycobacterium tuberculosis.* We selected soluble and full-length mutants of the recalcitrant Rv0113 protein using hybrid GFP insertion reporters with progressively increasing stringency during successive rounds of evolution.

## Results

### Construction of GFP protein insertion circular permutants

**Figure 3:**
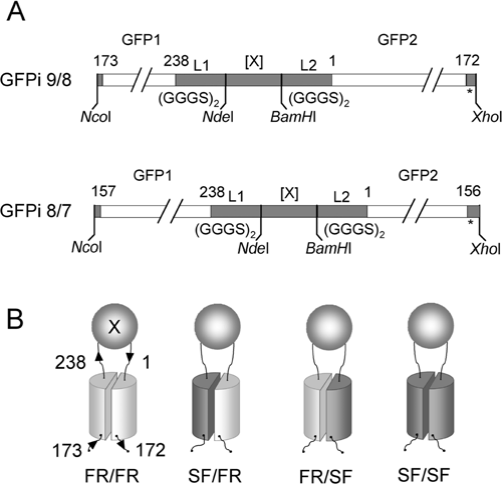
GFP insertion constructs. (A) GFP insertion constructs (GFPi) contain *Nde*I and *BamH*I cloning sites for accepting test proteins (X) between the C and N-termini of a circular permutant of GFP. Flexible amino acid linkers L1 and L2 GGGSGGGS separate the protein of interest from the two GFP domains, (GFP1 and GFP2). GFPi 9/8 starts at amino-acid position 173 (beginning of beta strand 9) and ends at amino-acid position 172 (end of beta strand 8) of GFP (*top*). GFP insertion 8/7, starts at amino-acid 157 (beginning of strand 8), and ends at amino-acid 156 (end of strand 7) (*bottom*). (B) Chimeric GFP insertion constructs of folding reporter GFP (*light gray*) and superfolder GFP (*dark gray*) scaffoldings (FR = folding reporter GFP, SF = superfolder GFP).

We previously described a series of rationally designed GFP circular permutants which start at an internal amino acid in GFP, continue to the C-terminus of natural GFP, connect to the N-terminus of natural GFP with a GGGS linker, and continue to complete the circular permutant of GFP [13]. The most fluorescent constructs started at amino-acid positions 157 or 173 for either the folding reporter GFP or superfolder GFP. We used these GFP circular permutants as a starting point to create chimeric GFP insertion (GFPi) constructs, where two GFP fragments flank the protein of interest [29]. To receive the insert gene, the GGGS linker sequence between the native N and C-termini of the GFP template was replaced with a DNA cassette containing a cloning site for test proteins (Fig. 3A). The so-called “GFP insertion” reporters are numbered according to the number of the beta-strand corresponding to the circular permutant break-point in the native topology of the eleven beta strand GFP sequence [13]. For example, GFPi 9/8 designates the expressed protein fusion [GFP (amino-acids 173 to 238)]-L_1_-[X]-L_2_-[GFP (amino-acids 1 to 172)], where L_1_ and L_2_ are flexible (GGGS)_2_ linkers, X is the inserted protein, beta strand 9 starts at residue 173 and beta strand 8 ends at residue 172 (Fig. 3A). A similar circular permutant insertion topology was created for the GFP circular permuted variant 8/7 starting at residue position 157 (Fig. 3A). To generate chimeric GFP insertion reporters with intermediate levels of folding robustness, we combined GFP scaffolding fragments from folding reporter GFP and from superfolder GFP, respectively (Fig. 3B). The resulting GFPi 9/8_FR/SF construct contains five folding mutations from superfolder GFP, whereas GFPi 9/8_SF/FR contains only one folding mutation from superfolder GFP (Fig. S1). GFPi 8/7_FR/SF and 8/7_SF/FR, contain 4 or 2 superfolder GFP mutations, respectively (Fig. S2).

### Effect of proteins with varying folding robustness on the fluorescence of the GFP insertion fusions

**Figure 4:**
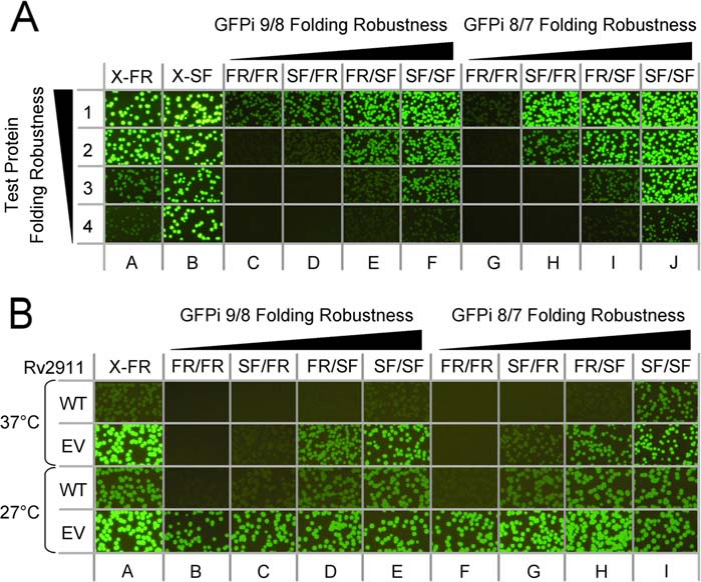
Sensitivity of various GFP insertion constructs (*columns A through J*) to protein misfolding. (A) Images of *E. coli* cells on plates expressing four test fusions proteins with decreasing folding robustness at 37°C. Protein #1 sulfite reductase (dissimilatory subunit) is fully soluble (*row 1*); protein #2 (translation initiation factor) is 70% soluble (*row 2*), protein #3 (3-hexulose 6-phosphate synthase) is 50% soluble (*row 3*), and protein #4 (polysulfide reductase subunit) is fully insoluble (*row 4*). Test proteins expressed as fusions with C-terminal folding reporter (X-FR) (*column A*), superfolder (X-SF) GFP (*column B*), or inserted in the four GFPi 9/8 reporters (*columns C–F*), or GFPi 8/7 reporters (*columns G–J*). Designations above each column designate the GFP variant from which each flanking GFP domain is derived (*see* *Fig. 3*). Exposure time is 2 s. (B) Fluorescent images of *E. coli* colonies expressing GFP reporter constructs of wild-type insoluble Rv2911 from *Mtb* (*rows marked WT*) and its evolved variant engineered using the C-terminal folding reporter GFP (*rows marked EV*) at two temperatures (27°C and 37°C). Columns and their designations correspond to the same GFP topologies indicated in Fig. 4a (*above*). Fluorescence was imaged after IPTG induction at 37°C and 27°C. Exposure time is 2 s.

To evaluate the folding robustness of all eight GFP circular permutant insertion reporters, the coding sequences of four *Pyrobaculum aerophilum* test proteins with known solubility and folding yield [30] (see Table 1), were each inserted between *Nde*I/*BamH*I restrictions sites of the cloning cassette (See Methods) (Fig. 4A). To compare these results with previously published data [13], the same test proteins were also fused to the N-terminus of the native topology folding reporter GFP or superfolder GFP. *E. coli* expressing the corresponding *P. aerophilum* GFP fusion proteins were plated on nitrocellulose membranes on selective LB-agar plates, incubated overnight at 32°C, and induced with isopropyl thiogalactoside (IPTG) for 4 hours at 37°C (see Methods), then colony fluorescence was imaged (Fig. 4A). Colony fluorescence decreased as the solubility of the inserted protein decreased for the C-terminal folding reporter GFP, as previously observed [1] (Fig. 4A, column A). In contrast, colonies expressing proteins fused to the C-terminal superfolder GFP were brightly fluorescent, even for the fully insoluble test protein #4 (polysulfide reductase subunit) (Fig. 4A, column B), consistent with the enhanced folding robustness of superfolder GFP [13]. Whole-cell fluorescence was lower for GFP insertion reporters compared to C-terminal GFP reporter fusions, especially for GFP insertion reporters derived from folding reporter GFP. Only the most soluble, well folded protein #1 (sulfite reductase) (Fig. 4A, row 1) could be detected using GFPi 9/8_FR/FR (Fig. 4A, columns C and G). As expected, GFP insertion reporters based on superfolder GFP (Fig. 4A, columns F and J) were far more tolerant to insertions than those based on folding reporter GFP, with fluorescence levels between the corresponding C-terminal GFP folding reporter (Fig. 4A, column A) and C-terminal superfolder GFP fusions (Fig. 4A, column B). Hybrid GFP insertion reporter constructs distinguished subtle differences in test protein folding robustness via the corresponding fluorescence levels. For example, GFPi 9/8_FR/SF (Fig. 4A, column E) and GFPi 8/7_FR/SF (Fig. 4A, column I) could detect differences in solubility between protein #3 (50% soluble as non-fusion) and protein #2 (70% soluble as non-fusion). The next less stringent vector, GFP insertion SF/FR, was able to detect protein #2 and still distinguish this candidate from the fully soluble protein #1 (Fig. 4A, column D and H). In contrast, cell fluorescence for these two proteins expressed as C-terminal GFP fusions was bright and indistinguishable (Fig. 4A, column A, rows 1 and 2), indicating that the conventional C-terminal GFP was unable to discriminate between the folding robustness of these two test proteins.

**Table 1:** Liquid culture fluorescence data of the GFP insertion constructs expressing four test proteins with progressively decreasing solubility at 37°C.

Topology^c^	Fraction soluble^b^	Test protein^a^			
		#1	#2	#3	#4
	Scaffolding^d^	1.00	0.70	0.50	0.00
		Normalized fluorescence^e^			
C-terminal	FR	8230	5800	435	110
C-terminal	SF	12425	12330	5580	1975
9/8 insertion	FR/FR	840	300	30	30
	SF/FR	1140	645	80	60
	FR/SF	2200	1775	430	180
	SF/SF	2575	1700	930	290
8/7 insertion	FR/FR	470	330	35	20
	SF/FR	2640	1800	105	90
	FR/SF	4550	3635	640	225
	SF/SF	3685	3700	2060	415

a Protein #1 sulfite reductase (dissimilatory subunit); protein #2 (translation initiation factor), protein #3 (3-hexulose 6-phosphate synthase), and protein #4 (polysulfide reductase subunit).

b Fraction of non-fusion protein soluble expressed in *E. coli* at 37°C, as determined by SDS-PAGE.

c Scaffolding topology of GFP folding reporter.

d Type of GFP domain used in reporter, SF = superfolder GFP, FR = folding reporter GFP.

e The measured fluorescence (488 nm excitation, 520 nm emission) normalized by dividing by the optical density at 600 nm.

To more accurately quantify whole cell fluorescence, the same cells expressing the four test proteins in all GFP reporters were grown and induced in liquid culture at 37°C. *E. coli* cell fluorescence was then measured and normalized by dividing whole cell fluorescence by the cell culture optical density (O.D. 600 nm) (Table 1). The normalized fluorescence values were in agreement with the apparent fluorescence of the colonies on membranes (compare Table 1 and Fig. 4A). The liquid culture fluorescence data also confirm the decrease in fluorescence as the stringency of the GFP insertion construct increases. For example, insertion of fully soluble protein sulfite reductase (protein #1) in the GFPi 9/8_SF/SF resulted in a three fold decrease in fluorescence levels compared to the original C-terminal folding reporter GFP fusion. We concluded that the new insertion GFP reporters are able to detect folding defects in different, unrelated test proteins that are not readily observed using the original C-terminal folding reporter.

### Distinguishing protein sequence variants with varying folding robustness

We tested the ability of the GFP insertion reporters to distinguish between the insoluble wild type Rv2911 (putative penicillin-binding protein) from *Mycobacterium tuberculosis* (*Mtb*) H37Rv and a soluble evolved variant of Rv2911, previously engineered using the conventional C-terminal folding reporter (Waldo et. al., unpublished results). Following directed evolution in the conventional C-terminal folding reporter GFP [1], [4], [31], sodium dodecyl sulfonate polyacrylamide gel electrophoresis (SDS-PAGE) of soluble and insoluble cell fractions indicated that the evolved variant of Rv2911 was fully soluble when expressed at 27°C, but insoluble at 37°C (data not shown), suggesting that latent folding defects in Rv2911 remained that became apparent at the more stringent expression temperature. To further evaluate the sensitivity of the insertion reporters, we measured the fluorescence of cells expressing the wild-type and the evolved protein Rv2911 as fusions with all eight GFP insertion reporters and also the C-terminal folding reporter GFP at two temperatures, 37°C and 27°C (Fig. 4B). At both temperatures, the less stringent GFP insertion reporters, GFPi 9/8_SF/SF, GFPi 9/8_FR/SF, GFPi 8/7_SF/SF, and GFPi 8/7_FR/SF 9/8, behaved similarly to the original C-terminal GFP folding reporter (Fig. 4B). However, we observed some striking differences using the more stringent GFP insertion reporters. At 37°C fluorescence of *E. coli* colonies expressing the evolved Rv2911 protein was barely detectable in GFP insertion vectors SF/FR and FR/FR (Fig. 4B, columns B, C, F and G) whereas the C-terminal folding reporter GFP fusions appeared very bright (Fig. 4B, column A), suggesting that a folding defect in Rv2911 still remains after evolution with the C-terminal GFP folding reporter. Such a latent defect would explain why directed evolution of Rv2911 using the less-stringent C-terminal folding reporter GFP produced a variant of Rv2911 capable of folding under more permissive expression conditions (27°C), but incapable of folding productively under more stringent conditions (37°C). Consistent with this interpretation, for most of the reporter topologies, colonies of *E. coli* expressing the fusions were fluorescent at 27°C (Fig. 4B, last row), but only the least stringent reporters were fluorescent at 37°C (Fig. 4B, second row, columns D, E, H, and I).

### Truncation artifacts arise when evolving Rv0113 wt using the C-terminal GFP reporter

**Figure 5:**
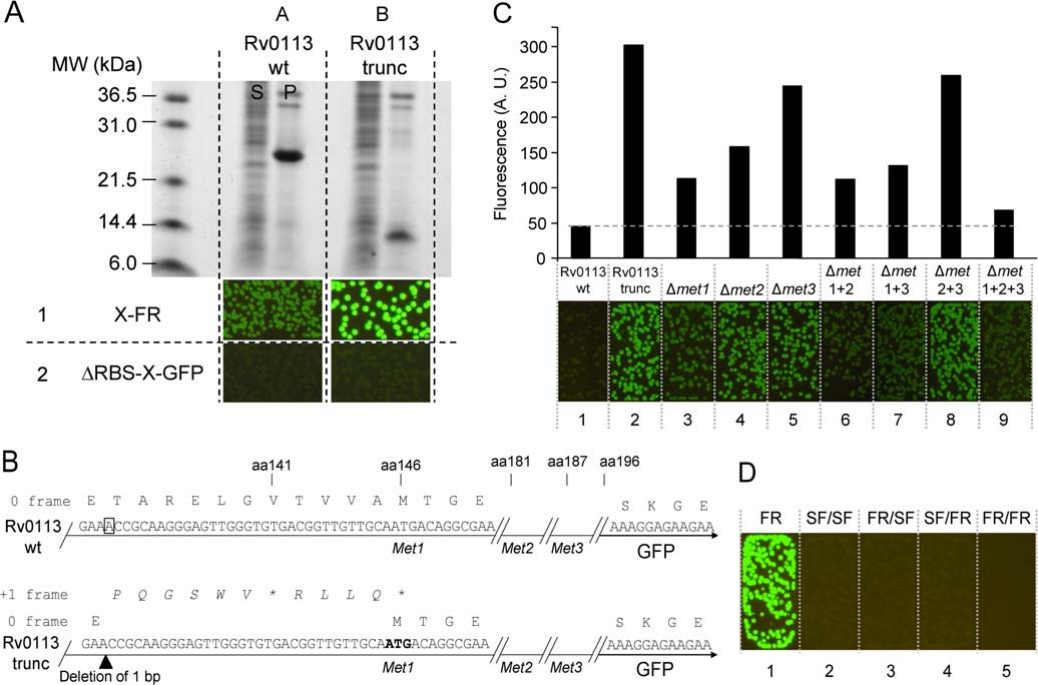
Characterization of a translation reinitiation artifact variant of Rv0113 isolated by directed evolution using C-terminal GFP FR. (A) Directed evolution of insoluble wild-type *Mtb* protein Rv0113 (*WT, row 1, column A*) produced a truncated protein variant that appeared bright as a GFP fusion (*EV, row 1, column B*). The same variant subcloned into a GFP fusion vector without a vector-encoded ribosome initiation sequence (ΔRBS-GFP) failed to produce bright colonies (*row2, column B*). Soluble fraction (S), pellet fraction (P). (B) DNA sequence of wild type Rv0113 (*top*) and truncated Rv0113 (*bottom*) GFP fusions. A single base pair deletion in the original frame at codon 134 (boxed in wild type Rv0113 sequence) (*top*), (arrow head below Rv0113 trunc sequence), (*bottom*) resulted in a new stop codon at amino acid 141. The frame-shifted residues (italic script above the Rv0113 sequence) (*bottom*). First methionine codon in the frame with GFP following the artifact (bold script) (*bottom*). Amino-acid positions shown above the sequences (C) Images of *E. coli* colonies expressing N-terminal polyhistidine fusions with C-terminal fused GFP for wild-type Rv0113 (*wt*), evolved truncated variant (*trunc*), and the evolved truncated variant with directed methionine→leucine substitutions at methionine 146 (*Δmet1*), methionine 181 (*Δmet2*), and methionine 187 (*Δmet3*). Double and triple methionine→leucine substitutions are also shown (*Δmet1 1+1, Δmet 1+3, Δmet 2+3, Δmet 1+2+3*). Exposure time is 2 s. Bar graph (*top*) of liquid culture fluorescence after expression of the same constructs in shake cultures at 37°C. Fluorescence values were normalized by dividing by optical density at 600 nm. (D) Fluorescence of *E. coli* colonies expressing Rv0113 truncated variant with original C-terminal GFP and indicated GFPi 9/8 reporters. The colonies expressing the C-terminal fusions (FR) are brightly fluorescent (*left image*). Colonies expressing the corresponding GFPi fusion constructs (*last four images*). Exposure time is 2 s.

We cloned Rv0113, a putative phosphoheptose isomerase from *Mtb*, from a cosMID library derived from *Mtb* H37Rv. We noted that when several independent clones of Rv0113 derived by PCR from the cosMID library were subsequently sequenced, each contained a single base deletion at bp 537 and a two base deletion at bp 572 (Sanger reference genome sequence numbering of *M. tb* H37Rv (http://www.doe-mbi.ucla.edu/TB/)). These lesions resulted in the replacement of a 13 amino acid residue block near the C-terminal end of the original protein sequence by a frame-shifted 12 amino acid block (Fig. S3) without the introduction of a stop codon, while keeping the first and last amino acids in the native frame. This lesion might have resulted from a random PCR error during cloning, or from a mutation in the cosMID library used as the template for the original PCR. The Rv0113 subclone, termed “Rv0113 wt”, was expressed as inclusion bodies in *E. coli* (Fig. 5A, column A). Although this is not the natural protein, it made a suitable test candidate for demonstrating directed evolution. The Rv0113 wt target gene was subjected to directed evolution using the C-terminal GFP reporter system [1], [4], [31]. After three rounds of DNA shuffling and selection at 37°C, one of the brightest optima (Fig. 5A, column B, row 1) was subcloned into a pET expression vector without GFP as previously described [1], and its solubility measured by SDS-PAGE. Surprisingly, this construct termed ‘Rv0113 trunc’ produced an insoluble truncated protein (Fig. 5A, column B), although the DNA was full-length (data not shown). The Rv0113 trunc was subcloned into a C-terminal GFP fusion vector lacking the upstream vector-encoded ribosome initiation sequence (Fig. 5A, ΔRBS_GFP vector) and expressed in *E. coli*. Colonies were non-fluorescent, suggesting that no internal translation had occurred from an alternate *de novo* ribosome binding site (Fig. 5A, column B, row 2). Examination of the DNA sequences revealed that a single base pair deletion had changed the frame of expression of the protein, leading to a premature stop codon at amino-acid 141 (Fig. 5B, bottom sequence). We hypothesized that at least one of the three methionine residues located near the new translation termination site in the shift frame of the single base deletion mutant (positions 146, 181, 187) might instead function as new translation reinitiation sites [32], [33] resuming expression of the C-terminus of Rv0113 and the fused GFP in the native frame (Fig. 5B). To test this hypothesis, we mutated each individual methionine residue into the closely-synonymous non-polar hydrophobic amino acid leucine which is not typically recognized as a translation initiation site. We analyzed the cell fluorescence after expression of the single, double and triple leucine mutants of Rv0113 trunc in the C-terminal GFP reporter vector at 37°C (Fig. 5C). Replacing methionine 146 with leucine (variant Δmet1, Fig. 5C, column 3) decreased whole-cell colony fluorescence more than did replacement of either methionine 181 or 187 by leucine (variants Δmet2 and Δmet3, Fig. 5C, columns 4 and 5). Moreover replacing both methionine 181 and 187 by leucine decreased cell fluorescence only slightly (Fig. 5C, Δmet2+3, column 8) relative to Rv0113 trunc (Fig. 5C, column 2). This suggested that methionine 146 was primarily responsible for reinitiating translation (Fig. 5C, column 3). Nonetheless, the reinitiation event seemed cooperative since simultaneously replacing all three methionine residues (146, 181, 187) by leucine residues (Δmet1+2+3) further reduced GFP fluorescence (Fig. 5C, column 9). To characterize the putative translation reinitiation products in greater detail, we used Talon® (*Clontech*) metal affinity resin (Methods) to bind proteins from soluble and urea-unfolded insoluble cell fractions from *E. coli* expressing the Rv0113 and various Rv0113 trunc constructs as N6HIS-X-GFP fusions. Since the GFP moiety of GFP fusions retains fluorescence when the GFP fusion is solubilized in 9M urea, and 6HIS-tagged proteins bind to Talon® resin in 9M urea, both the soluble and insoluble fractions could be examined for potential binding to Talon® resin. As expected, a full-length protein fusion with apparent molecular weight of ca. 55 kDal was observed in the Talon®-resin-bound urea-denatured insoluble cell fraction for Rv0113 wt as revealed by SDS-PAGE (Fig. S4). In contrast, the Rv0113 trunc variant and its triple methionine-to-leucine substituted variant produced only aberrant, low molecular-weight insoluble proteins capable of binding Talon® beads when denatured by urea as revealed by SDS PAGE (Fig. S4). These truncations still had an N-terminal polyhistidine tag and so did not come from internal *de novo* ribosome binding sites. On the other hand, fluorescence measurements of soluble extracts indicated that significant amounts of GFP were produced from the bright Rv0113 trunc fusion clones (Fig. S4), though below the level readily detectable by SDS PAGE (Fig. S4). Many proteins derived from translation reinitiation are expressed at significantly lower levels relative to the protein derived from the *de novo* ribosome binding site of the requisite upstream open reading frame [32], [33]. These fluorescent products did not bind Talon®, so likely arose from internal translation sites (Fig. S4). As expected, we also observed soluble GFP fluorescence from the Rv0113 trunc variant with substituted methionine residues, but in lower amounts (Fig. S4). Taken together, these observations support the notion that, in the mutant of Rv0113 derived from the directed evolution using the C-terminal GFP folding reporter, two aberrant protein products are produced: a C-terminal truncated Rv0113 variant with an intact N-terminus derived from the native frame of the Rv0113 coding sequence but containing a frame-shift and stop codon, and a shift-frame translation reinitation product from near the C-terminus of the Rv0113 mutant gene in-frame with the downstream fused GFP. Apparently the reinitiation products lead to bright fusions in the context of the original C-terminal GFP folding reporter (Fig. 5A, column B, row 1, and Fig. 5D, column 1). To assess if such reinitiation artifacts would have been detected using the new GFP insertion reporters, we subcloned the bright Rv0113 trunc variant into the set of four 9/8 GFP insertion reporters. No fluorescence was observed after expression of the fusion protein from the four vectors at 37°C (Fig. 5D, columns 2–5). This suggested that the insertion topology of the new GFP reporters could useful for discriminating against artifacts that result from translation reinitiation.

### Directed evolution schema of protein folding in GFP insertion vectors and application to Rv0113 wt

**Figure 6:**
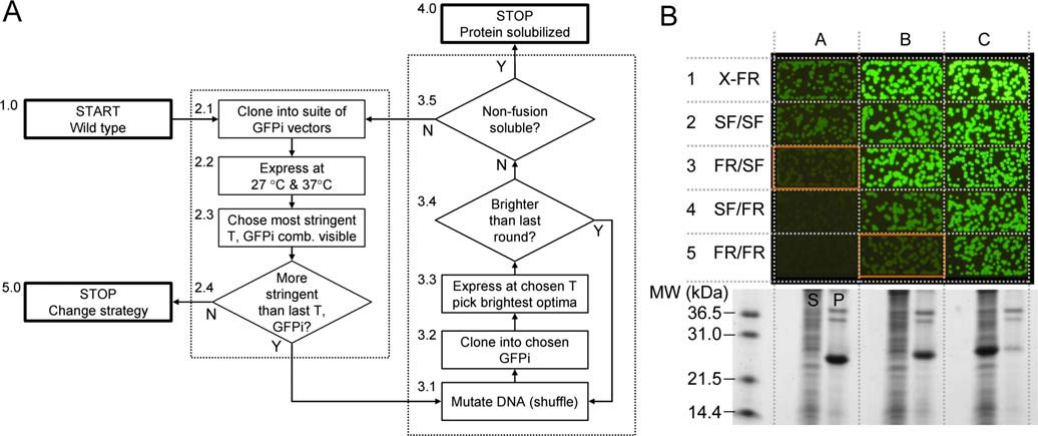
Directed evolution of protein folding using GFP insertion reporters and application to Rv0113. (A) General strategy for improving protein folding from multiple reporters with increased stringency (*see text for detailed explanation*). (B) Directed evolution of Rv0113 variant in GFPi 9/8 vector family. Constructs evaluated in X-FR (*row 1*) and the four GFPi 9/8 constructs (*rows 2–5*). Images of *E. coli* colonies expressing wild type Rv0113 in the indicated reporters (*column A*), brightest optimum after three round of evolution in GFPi 9/8_FR/SF expressed in the indicated reporters (*column B*), and brightest optimum after two additional rounds in GFPi 9/8_FR/FR (*column C*). All constructs expressed at 37°C. Exposure time is 2 s. The SDS-PAGE gel (*bottom*) shows soluble (*S*) and pellet (*P*) fractions for each variant (*columns A, B, C*) cloned without the fused GFP domains in a C-terminal polyhistidine pET vector, expressed in shake cultures at 37°C.

We designed a general strategy that combines classical mutagenesis methods with the suite of GFP insertion reporters for screening protein variants with improved folding characteristics (Fig. 6A). The protocol starts with the wild type gene (Fig. 6A, Step 1.0), in this case Rv0113 wt. Next, the GFP insertion vector with the appropriate stringency is chosen. The starting gene is cloned into the desired suite of GFP insertion vectors (typically the four 9/8 or 8/7 vectors) (Fig. 6A, Step 2.1). We chose GFPi 9/8 insertion reporters in this case because they appeared slightly more stringent than the GFPi 8/7 series (Table 1). Next the panel of GFP insertion vectors containing the gene(s) is expressed at two temperatures, i.e. 27°C or 37°C, and the fluorescence evaluated (Fig. 6A, Step 2.2). The combination of vector/temperature giving the minimum detectable fluorescence signal is chosen for the first round of evolution (Fig. 6A, 2.3). The GFPi 9/8_FR/SF reporter was chosen for the first round of evolution based on the fluorescence of the wild-type Rv0113 gene expressed within the four GFPi 9/8 reporters (Fig. 6B, column A, row 3). Following the flow chart Fig. 6A, Steps 3.1–3.3, libraries of shuffled Rv0113 were cloned in the insertion site of GFPi 9/8_FR/SF. About 20,000 clones were plated and expressed at 37°C, and 96 of the brightest clones were picked. During the first two rounds, the overall colony fluorescence levels of the top 16 optima continued to increase relative to the previous round (Fig 6A, Step 3.3), and the 16 optima were recombined after each round of evolution for a new cycle of shuffling and selection in the same GFP insertion reporter (GFPi 9/8_FR/SF). At the third round, the fluorescence of the GFP insertion fusions did not improve (Fig. 6A, Step 3.3) and following the flowchart Fig. 6A, Step 3.4, we assessed the solubility of one of the best optima by SDS-PAGE. The variant subcloned without the fused GFP was expressed as a full-length protein but was insoluble (Fig. 6B, see SDS gel under column B). Despite the low solubility of this Rv0113 variant expressed without the fused GFP domains, the GFPi 9/8_FR/SF fusion appeared brightly fluorescent (Fig. 6B, column B, row 3). Following the flowchart (Fig. 6A, Step 3.5), since the subcloned non-fusion Rv0113 protein was insoluble, we screened for a more stringent vector to continue addition rounds of directed evolution. We tested the current Rv0113 optimum in all four GFPi 9/8 vectors (Fig. 6A, Steps 2.1–2.4). Colonies expressing the Rv0113 optimum in the more stringent reporters GFPi 9/8_SF/FR and GFPi_9/8 FR/FR (Fig. 6B, column B, row 4 and row 5) were clearly fainter than colonies expressing the Rv0113 optimum in the 9/8_FR/SF vector (Fig. 6B, column B, row 3). Based on these observations, we screened the next pool of mutants in the most stringent vector GFPi 9/8_FR/FR. After two rounds of evolution using the GFPi 9/8_FR/FR reporter, one performed at 27°C and a second round at 37°C (Fig. 6A, Steps 3.1–3.4), the library of clones appeared homogenously bright (Fig. 6B, column C, row 5) with little or no variation in the levels of fluorescence throughout the population of colonies.

Following the flowchart (Fig. 6A, Step 3.5), DNA coding for the Rv0113 variant of a single bright optimum colony was subcloned into a C-terminal polyhistidine pET vector as previously described [1], [4]. The Rv0113 was fully soluble and full-length as determined by SDS-PAGE (Fig. 6B, bottom inset, column C). DNA sequencing of several optima clones obtained after evolution in GFPi 9/8_FR/SF or after continued evolution using the more stringent GFPi 9/8_FR/FR, revealed consensus mutations V12E, N54D, G109R, and S176F (Fig. S5). The mutation N54D occurred only after the additional rounds in the most stringent vector GFPi 9/8_FR/FR at 37°C, and is likely the key to the improved solubility relative to the first rounds in GFPi 9/8_FR/SF reporter (Fig. S5).

We were able to successfully evolve a soluble, well-expressed variant of Rv0113 using the GFP insertion reporters. If further improvement in folding had been desired, the current pool of optima would again be evaluated in the suite of GFP insertion vectors at two temperatures (Fig. 6A, Steps 2.1–2.4) and the directed evolution cycles continued with the more stringent conditions. Optima with improved folding may be then screened for solubility using the split-GFP solubility reporter [34]. If solubility does not improve or no further increase in stringency is possible, an alternative strategy may be indicated (Fig. 6A, Step 5.0) such as screening for co-expression with a folding partner, or alternative hosts, for example.

## Discussion

Previously described protein folding assays used C-terminal fused fluorescent proteins such as jellyfish fluorescent protein [1], [4] or reef coral fluorescent proteins [35] to report on the folding robustness of upstream fused polypeptides. Heddle et. al screened four reef coral proteins using a panel of test proteins of known solubility and determined that a single fluorescent protein, ZsGreen, provided the best compromise between detection (overall fluorescence and signal-to-noise) and dynamic range (difference in fluorescence of cells expressing ZsGreen fusions to the best-folding and poorest-folding test proteins). Since the other coral proteins tested varied significantly in folding properties, sequence, and color, it is unclear whether they might nonetheless be useful alternatives to tailor reporter sensitivity to misfolding by a particular fusion protein.

In contrast, we have generated a panel of eight folding reporters all derived from GFP, each with a distinct sensitivity to test protein misfolding, by changing the circular permutant start site in the GFP scaffolding and number of superfolder mutations [13]. Since they are all derived from very closely related variants of the same basic fluorescent protein scaffolding, our panel of GFP insertion reporters provides a well-characterized and graded sensitivity to misfolding by test proteins cloned between the N- and C-termini of the reporter. Our results show that fluorescence of GFP insertion reporters containing different test proteins (Fig. 4A) reflects the robustness of the GFP variant scaffolding from which the reporters are derived (Fig. 3B). The more stringent GFP insertion constructs derived from folding reporter GFP should be useful for evolving proteins with subtle folding defects that would evade detection by C-terminal GFP folding reporter (Fig. 4A and Fig. 4B). Although C-terminal fused superfolder GFP is relatively insensitive to misfolding by upstream polypeptides [13] (Fig. 4A, column B), circular permutation is sufficiently destabilizing such that insertion-type reporters based on superfolder GFP (Fig. 4A, column F and column J) behave similarly to the C-terminal GFP folding reporter (Fig. 4A, column A). Consequently, insertion folding reporters based on superfolder GFP are useful for screening very poorly folded proteins (Fig. 4A). Proteins with intermediate folding defects are efficiently screened by using chimeric GFPi constructs that combine folding reporter GFP and superfolder modules (Fig. 4A).

For a given circular permutant topology, i.e. either GFPi 8/7 or GFPi 9/8 (Fig. 3a), sensitivity of the four reporters to test protein misfolding is well-correlated with the number of superfolder mutations in the corresponding GFP domains (Fig. 4A, see also Fig. S1). Cell fluorescence of GFPi 8/7_FR/SF and SF/SF fusions was generally brighter than the corresponding 9/8 variants (See Table 1 and Fig. 4A). This is consistent with our previous observation that the parent GFP circular permutants (containing no test insert) starting at 157 (corresponding to 8/7) are intrinsically more fluorescent than those starting at 172 (corresponding to 9/8) [13]. Alternatively, the GFP insertion 8/7 FR/SF variants might be less sensitive to test protein folding interference due to the shorter length of the 8/7 reporter C-terminal GFP domain compared to the longer 9/8 C-terminal GFP domain (Fig. 3A). We hypothesize that the GFP folding reporters function by a so-called ‘folding interference’ mechanism, involving the formation of non-productive, non-fluorescent trapped folding intermediates of the GFP moiety and fused test protein domains. In this model, a larger GFP domain might exhibit a more complex folding trajectory than a shorter GFP domain, increasing the probability for interaction between the folding intermediates of the GFP domain and the upstream (N-terminal) domains of the protein of interest. These hypotheses are being tested in our laboratories.

Application of the C-terminal folding reporter GFP and insertion circular permutants to several test proteins demonstrated that the new generation of insertion reporters can detect misfolding defects that were not detectable using the original C-terminal folding reporter GFP (Fig. 4). For example, the evolved Rv2911 is insoluble at 37°C, even though the C-terminal folding reporter GFP fusion is brightly fluorescent (Fig. 4B). In contrast, fusions of the evolved Rv2911 to the high-stringency insertion vectors 8/7_FR/FR and 9/8_FR/FR are not fluorescent (Fig. 4B). This indicates that evolution of Rv2911 in the C-terminal folding reporter GFP produced species with temperature sensitive folding defects, i.e they fold productively at 27°C but not at 37°C. One would expect that additional cycles of molecular evolution in GFP insertion vectors with increasing stringency would likely produce a more robust and soluble Rv2911 variant. The folding trajectory of the GFP insertion provides a new basis for discriminating truncation artifacts generated during a mutagenesis process. We have demonstrated that evolution of Rv0113 as a C-terminal fusion to GFP folding reporter produced a truncation artifact while reinitiating expression of full-length GFP (Fig. 5A). This translation reinitiation event appeared to be primarily dependent on nearby methionine residue located 10 bp downstream from the stop codon in artifact Rv0113 and in the GFP coding frame. This internal translation site did not function as a *de novo* ribosome binding site. Instead, translation of the truncated peptide appeared to be dependent on the functional upstream vector-encoded ribosome binding site 5′ to the Rv0113 open reading frame, an example of translation reinitiation [32], [33]. When the artifact was expressed in any of the GFPi vectors, *E. coli* colonies were not detectably fluorescent (Fig. 5D). As previously described in the literature, translation reinitiation occurs in at least 5% of in-frame expressed proteins [36]. In the GFP insertion topology, it is less likely that sufficient amount of folded downstream GFP domain is produced to reconstitute the full-length GFP in minimum detectable quantities, especially if at least one of the expression products fused to a GFP fragment is poorly folded (Fig. 2). Similarly, one would expect that other types of artifacts such as *de novo* cryptic ribosome-binding site(s) will produce two separate translation products that are less likely to complement and fluoresce, particularly if one of the two GFP fragments is attached to a poorly folded target protein domain, such as Rv0113.

We have devised a new strategy for performing directed evolution experiments (Fig. 6A) by increasing stringency of screening while enriching the population in sequences with a desired phenotype, roughly analogous to increased stringency/cycle frequently used in display experiments for selecting high affinity binders [20], [37]–[39]. Instead of modifying the conditions in which the selection is performed (more stringent washes in the case of phage display) we used a panel of GFPi vectors exhibiting different susceptibility to test protein folding interference (Fig. 4). For very poorly-folded proteins, one can start with a low-stringency insertion vector, such as 9/8_FR/SF or GFPi 9/8 SF/SF, to begin the directed evolution trajectory. Further cycles of evolvtion can be performed if necessary, using increasingly stringent GFPi reporters. At the end of the evolution strategy, the most stringent insertion reporters enable selection of only the best folded variants. Following the scheme outlined in Fig. 6A, we successfully evolve a soluble variant (Fig. 6B) of a putative phosphoheptose isomerase (Rv0113) from *Mtb*.

Taken together, the suite of eight GFPi vectors provides a wide range of sensitivity to test protein misfolding, and the insertion topology provides better discrimination against internal translation products relative to the original C-terminal GFP folding reporter. The GFP insertion topology folding reporters should be useful additions to the tools available for measuring and engineering protein folding and solubility.

## Materials and Methods

### Construction of GFP insertion reporters

A cloning cassette was synthesized by PCR extension of the GFP fragments. Specific primers P1 (5′-CCCGCCCGACCCACCGCCTTTGTAGAGCTCATCCATGCCATG-3′), P2 (5′-TGAACCGCCACCCATATGGGAGCCCCCGCCCGACCCACCGCC-3′) were used for extension of the 3′-end of fragment GFP1. Primers P3 (5′-GTGGAGGGTCAGGGGGCGGATCAAGCAAAGGAGAAGAACTTTT-3′), P4 (5′-CATATGGGTGGCGGTTCAGGATCCGGTGGAGGGTCAGGGGGCG-3′) were used to extend the 5′-end of fragment GFP2. Reassembly between homologous sequences of P2 and P4 lead to the full-length amplification of the NH_2_-*GFP1*-GGGSGGGSHM-GGGS-GSGGGSGGGS-*GFP2*-COOH sequence. GFP 1 corresponds to amino-acid residues 174–238 and GFP2 to amino-acid residues 1–173 in GFPi 9/8 topology. The 8/7 topology consists of GFP1: 157–238 and GFP2: 1–156. A frame shift stuffer (FS1) (5′-CATATGTAATTAATTAATTGGATCC-3′) was inserted between *Nde*I/*BamH*I to guard against false-positives arising from undigested plasmid. The whole cassette was inserted using *Sph*I/*Kpn*I of the GFP folding reporter vector [1]. To create hybrid reporters_,_ GFP domains 1 and 2 were exchanged from one variant to another using *Sph*I/*Nde*I and *BamH*I/*Kpn*I restriction sites, respectively.

### Cloning of test inserts in GFP insertion

Four proteins from *Pyrobaculum aerophilum* (See reference [30] for complete data) were selected. Protein #1, a sulfite reductase (dissimilatory subunit), is fully soluble; protein #2, a translation initiation factor, is 70% soluble; protein #3, a 3-hexulose 6-phosphate synthase, is 50% soluble; protein #4, a polysulfide reductase subunit is fully insoluble. *Mtb* Rv0113 (putative phosphoheptose isomerase) and Rv2911 (putative penicillin-binding protein) wild-type and mutant DNAs were restricted using *Nde*I and *BamH*I restriction enzymes (NEB) and gel purified by agarose electrophoresis. C-terminal GFP folding reporter [1], GFP insertion reporters and ΔRBS_GFP vectors were all restricted using *Nde*I and *BamH*I and dephosphorylated using 0.2 µl of calf intestinal alkaline phosphatase (CIP) (NEB) to receive inserts. Ligations were performed using 3.8 µl of DNA insert, 1.0 µl of vector, 1.0 µl of 5× T4 DNA ligase buffer (Invitrogen), and 0.3 µl of T4 DNA ligase 400 U/µl (NEB). 2.0 µl of ligated product was transformed in 40.0 µl of *E. coli* BL21(DE3) strain made chemically competent.

### Expression screening of GFP fusions on nitrocellulose membranes

Single clones expressing test proteins as GFP fusions (insertion topology or C-terminal GFP) were grown in liquid culture in Luria-Bertani (LB) media plus kanamycin (35 µg/ml) and frozen in LB, 20% glycerol at OD_600nm_ = 1.0. For single colony dilution on membrane, 1.0 OD (600 nm) frozen stocks were diluted using two 400-fold serial dilutions in 1 ml LB. 50 µl was used to plate cells on a 4×8 grids printed on 130 mm diameter nitrocellulose membranes on selective LB/agar Bauer plates containing 35 µg/ml kanamycin. After overnight growth at 32°C, the membrane was moved onto a LB/Agar plus kanamycin (35 µg/ml) and 1 m*M* isopropyl-β-D-thiogalactopyranoside (IPTG), and incubated for 4 h at 37°C or 27°C. After induction, the colonies were illuminated using an Illumatool Lighting System® (LightTools Research, Encinitas, CA ) equipped with a 488 nm excitation filter, and photographed with a DC290 digital camera (Kodak) through a colored glass filter (520 nm long pass, LightTools Research).

### Metal affinity resin binding assays of Rv0113 N6HIS GFP fusions

Expression, lysis, and SDS-PAGE analysis of insoluble wild type Rv0113, truncated Rv0113 selected by evolution using C-terminal GFP reporter, and truncated Rv0113 with methionine-to-leucine substitutions (Δmet1+2+3) cloned as N6HIS-Rv00113-GFP fusions was performed as previously described [1]. Small scale binding assays were performed in 1.5 ml eppendorf microcentrifuge tubes. 50 µl of a 50% (v/v) slurry of Talon® (His)6 affinity resin beads (Clontech, Palo Alto, CA) in 100 mM Tris HCl pH 7.4, 0.1 M NaCl, 10% glycerol v/v (TNG buffer) was incubated with 50 µl of soluble protein extract. After centrifugation, the unbound fraction (U) was saved and the beads were washed twice with 500 µl of TNG buffer. Excess supernatant was removed by pipetting, and then 50 µl of 2×SDS buffer was added to the dried beads. The beads were heat-denatured in an MJR Research PCR machine and resolved by SDS-PAGE. To analyze protein binding from pellets, inclusion bodies were washed with 5 volumes of TNG and unfolded in TNG buffer containing 9 M urea. 50 µl of the urea-unfolded solubilized pellet was mixed with Talon® beads prewashed with TNG+9M urea. Talon® binding assay of unfolded insoluble fractions was performed similarly as the soluble assay above except that 9M urea was included in the buffer(s) throughout the experiment.

### Directed evolution of *M. tuberculosis* targets

Mutant library construction and screening was performed as previously described [1], [4]. For Rv0113 engineering, GFP insertion reporters FR/SF and SF/FR were used instead of the C-terminal folding reporter GFP (See [34] for a detailed protocol).

### Methionine substitutions in Rv0113 artifact

A pET_C6HIS plasmid containing the artifactual Rv0113 variant was used for construction of single methionine (ATG) to leucine (CTG) substitutions (Δmet mutations) using overlap extension PCR. Two PCR were performed with each one of the mutagenic primers and the corresponding vector specific primer. Single PCR fragments gel-purified and assembled in a subsequent PCR using vector specific primers to generate the full-length mutant DNA. Construction of Δmet1+Δmet2 Rv0113 trunc and Δmet1+Δmet3 Rv0113 trunc substitutions used single met1→leu variant as template. Finally, Δmet1+Δmet2 Rv0113 trunc plasmid template was used to generate the Δmet1+Δmet2+Δmet3 Rv0113 trunc variant and mutagenic substitution was introduced at Δmet3.

### Fluorescence measurements from liquid cultures

GFP fusions (insertion or C-terminal) were grown and expressed in liquid culture as described [1]. Cell pellets were resuspended in 100 mM Tris HCl pH 7.4, 0.1 M NaCl, 10% glycerol v/v (TNG buffer) and diluted 5 fold in TNG. Fluorescence was measured in white 96-well assay plates with low fluorescence background (Nunc-Immuno™) using a FL600 Microplate Fluorescence Reader (Biotek, Winooski, VT). The background fluorescence of a blank sample (*E. coli* lysate expressing an irrelevant protein) was subtracted from final fluorescence values. Cell density of each dilution was assessed by measuring optical density at 600 nm. Fluorescence was normalized by dividing by cell density.

## Supporting Information

Figure S1 Schematic diagram of the four GFPi 9/8 insertion vectors. Constructs start at amino-acid 173 (beginning of beta-strand 9 of GFP) and end at amino-acid 172 (end of beta-strand 8 of GFP). Stringency decreases as the number of superfolder mutations increase going from FR/FR to SF/SF. Folding mutations from superfolder GFP are shown in bold.
(0.34 MB TIF)

Figure S2 Schematic diagram of the four GFPi 8/7 insertion vectors. These constructs start at amino-acid 157 (beginning of beta-strand 8 of GFP) and end at amino-acid 156 (end of beta-strand 7 of GFP). Stringency decreases as the number of superfolder mutations increase going from FR/FR to SF/SF. Folding mutations from superfolder GFP are shown in bold.
(0.32 MB TIF)

Figure S3 Amino-acid sequence of Rv0113 wild-type Sanger Database reference sequence (Rv0113 TB DB) (http://www.doe-mbi.ucla.edu/TB/) and cloned Rv0113 (*Rv0113 Cloned*). DNA sequencing of the cloned Rv0113 revealed a single base deletion at bp 537, and a two-base deletion at bp 572 relative to the original Rv0113 reference sequence (*black boxes indicated by arrows*). This led to the replacement of 13 amino acids near the C-terminus of the original protein (*pink box*) with a frame-shifted peptide (*red box*) in the cloned Rv0113. This resulted in a net single amino acid deletion keeping the first and last amino acids in the native frame with no stop codon.
(1.06 MB TIF)

Figure S4 (A) SDS-PAGE of Talon® resin-binding of soluble and insoluble fraction of wild type Rv0113 type (*(Rv0113 wt)-GFP*), truncated Rv0113 (*(Rv0113 trunc)-GFP*), and truncated Rv0113 with three methionine-to-leucine substitutions (*(Rv0113 truncΔmet)-GFP*) variants as N6HIS-X-GFP fusions. Soluble extracts were bound to Talon®beads under native conditions (N), whereas insoluble pellets were unfolded in 9M urea and bound to Talon® resin under denaturing conditions in 9M urea (D). Total extract (T), unbound protein (U) and bound protein (B). (B) Fluorescence of corresponding samples, total extract (T), unbound protein (U) and bound protein (B), measured using a BioTEK plate reader.
(2.93 MB DOC)

Figure S5 Amino acid sequence alignment of Rv0113 starting variant and brightest mutants from successive rounds of directed evolution using the GFP insertion reporters. DNA sequences of five optima obtained after three rounds (Rd3) in the least stringent FR/SF reporter (*shown below the dotted line in each set*). Round 5 DNA sequences of six optima obtained after taking the round 3 optima through two additional cycles in the most stringent FR/FR vector (Rd5) (*shown above the dotted line in each set*). Mutations found in some optima of round 3 were highly enriched after round 5 (*orange highlight*). One additional mutation N54D appeared only after round 5 and is correlated with increased solubility of the new mutants (*yellow highlight*).
(0.70 MB TIF)
