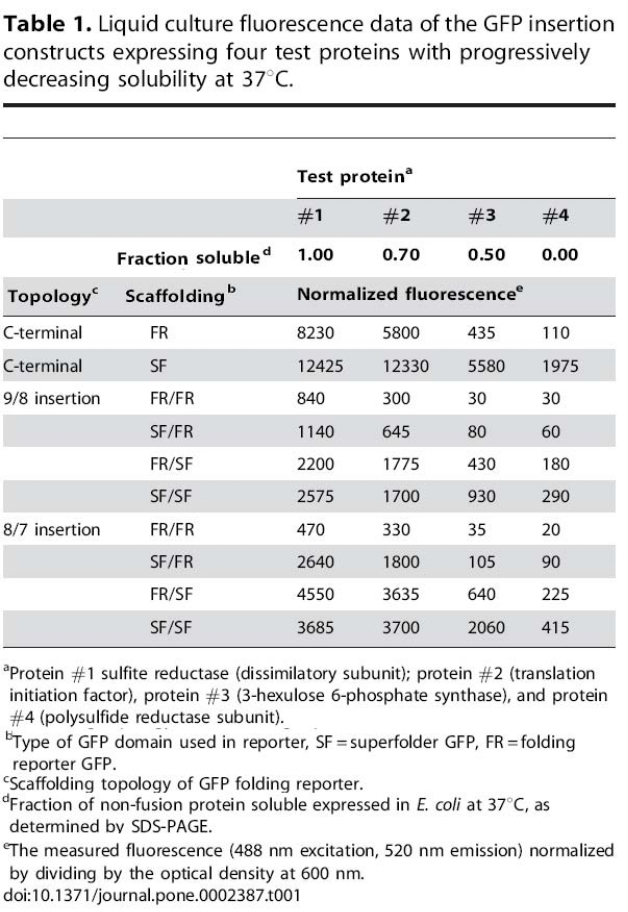# Correction: New Molecular Reporters for Rapid Protein Folding Assays

**DOI:** 10.1371/annotation/acff33c4-bf92-4edb-ac57-53154ea38ab4

**Published:** 2008-06-13

**Authors:** Stéphanie Cabantous, Yvonne Rogers, Thomas C. Terwilliger, Geoffrey S. Waldo

The headings in Table 1 are incorrect. Please view the correct table here:

**Figure:**