# Supplementary figures and images for: New Molecular Reporters for Rapid Protein Folding Assays
Source: PLoS One. 2008 Jun 11;3(6):e2387. doi: 10.1371/journal.pone.0002387 (PMC2408556; doi:10.1371/journal.pone.0002387)

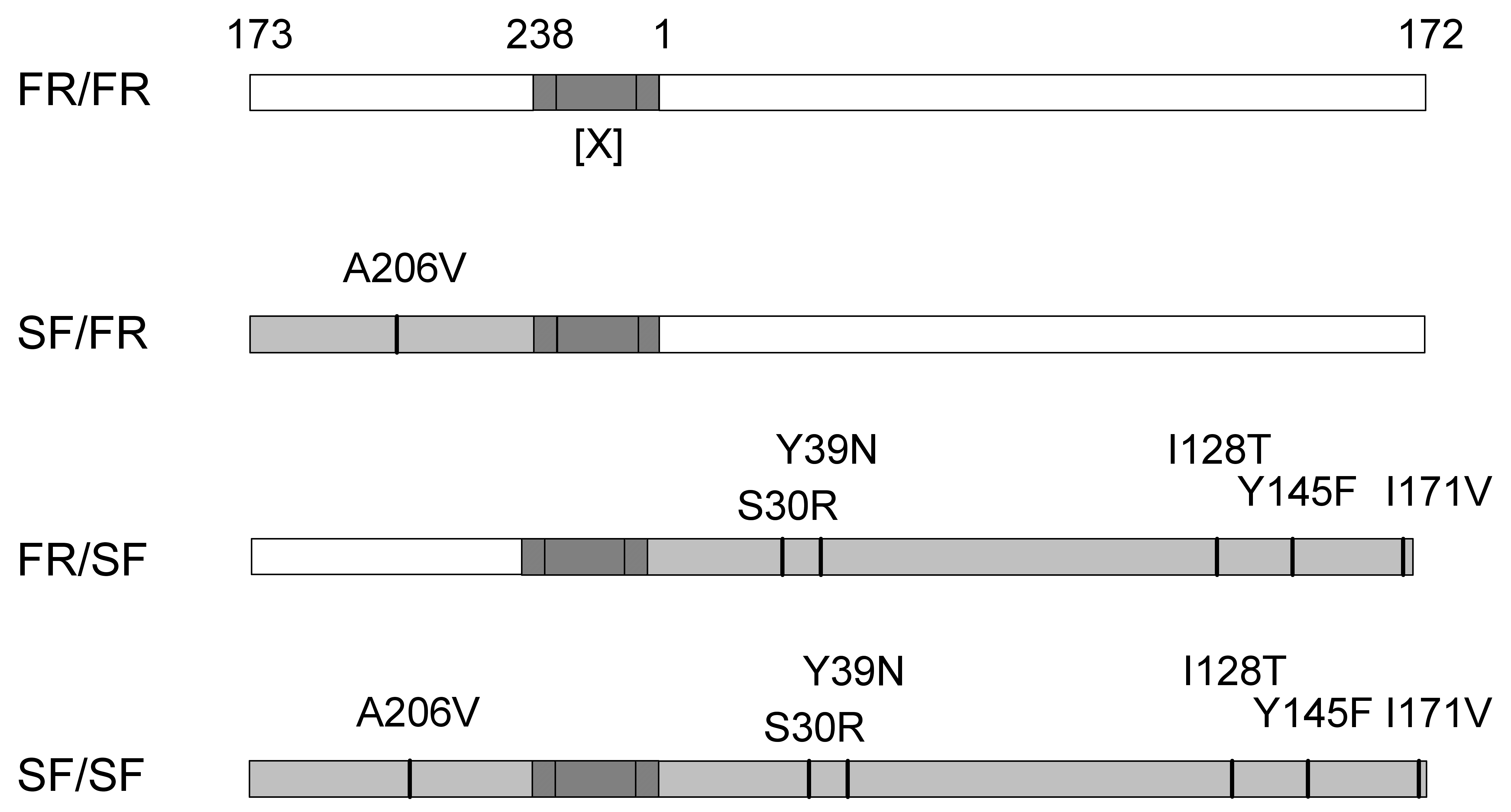

Supplement: Figure S1 — Schematic diagram of the four GFPi 9/8 insertion vectors. Constructs start at amino-acid 173 (beginning of beta-strand 9 of GFP) and end at amino-acid 172 (end of beta-strand 8 of GFP). Stringency decreases as the number of superfolder mutations increase going from FR/FR to SF/SF. Folding mutations from superfolder GFP are shown in bold. (0.34 MB TIF) [file pone.0002387.s001.tif]

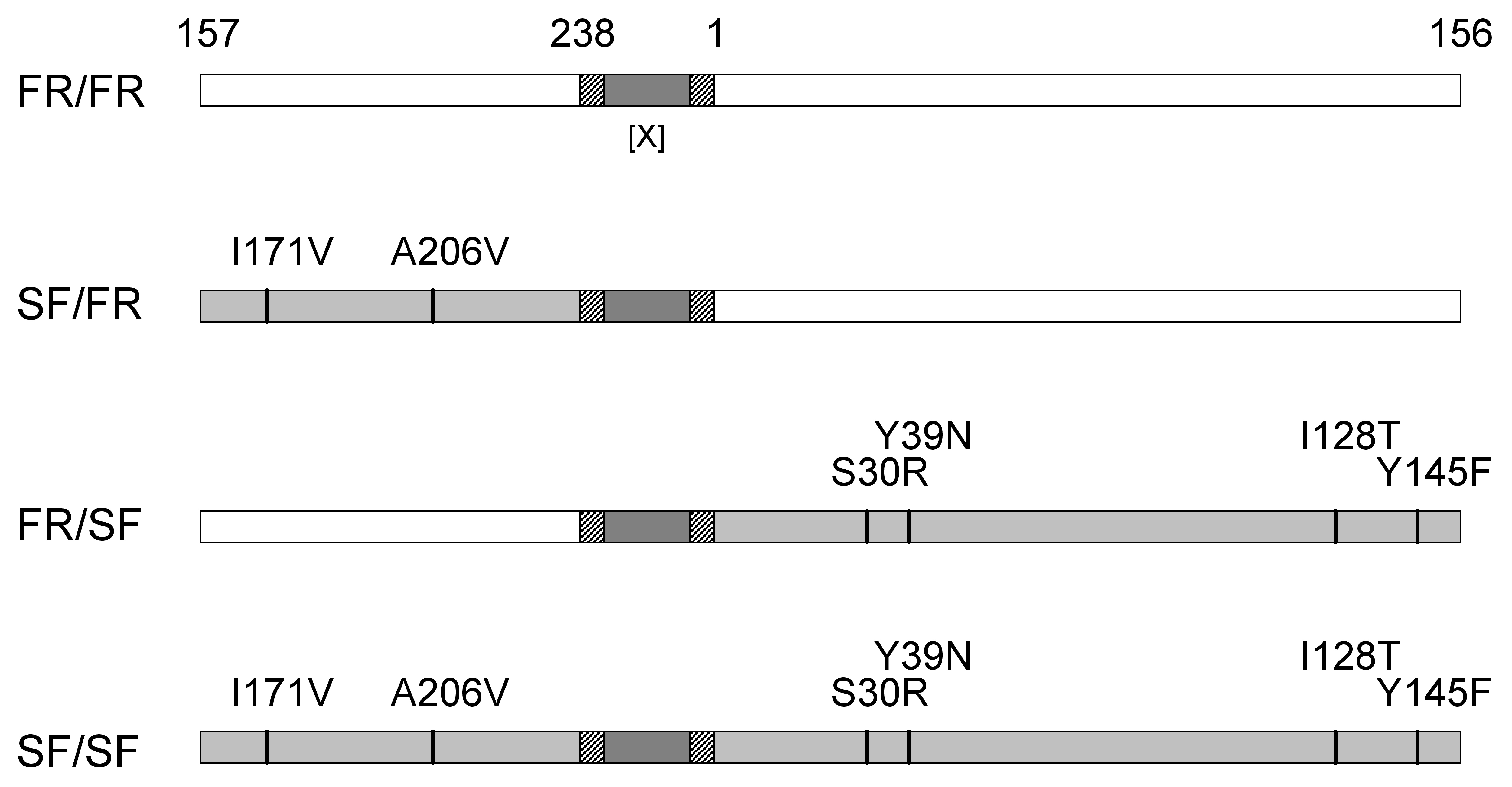

Supplement: Figure S2 — Schematic diagram of the four GFPi 8/7 insertion vectors. These constructs start at amino-acid 157 (beginning of beta-strand 8 of GFP) and end at amino-acid 156 (end of beta-strand 7 of GFP). Stringency decreases as the number of superfolder mutations increase going from FR/FR to SF/SF. Folding mutations from superfolder GFP are shown in bold. (0.32 MB TIF) [file pone.0002387.s002.tif]

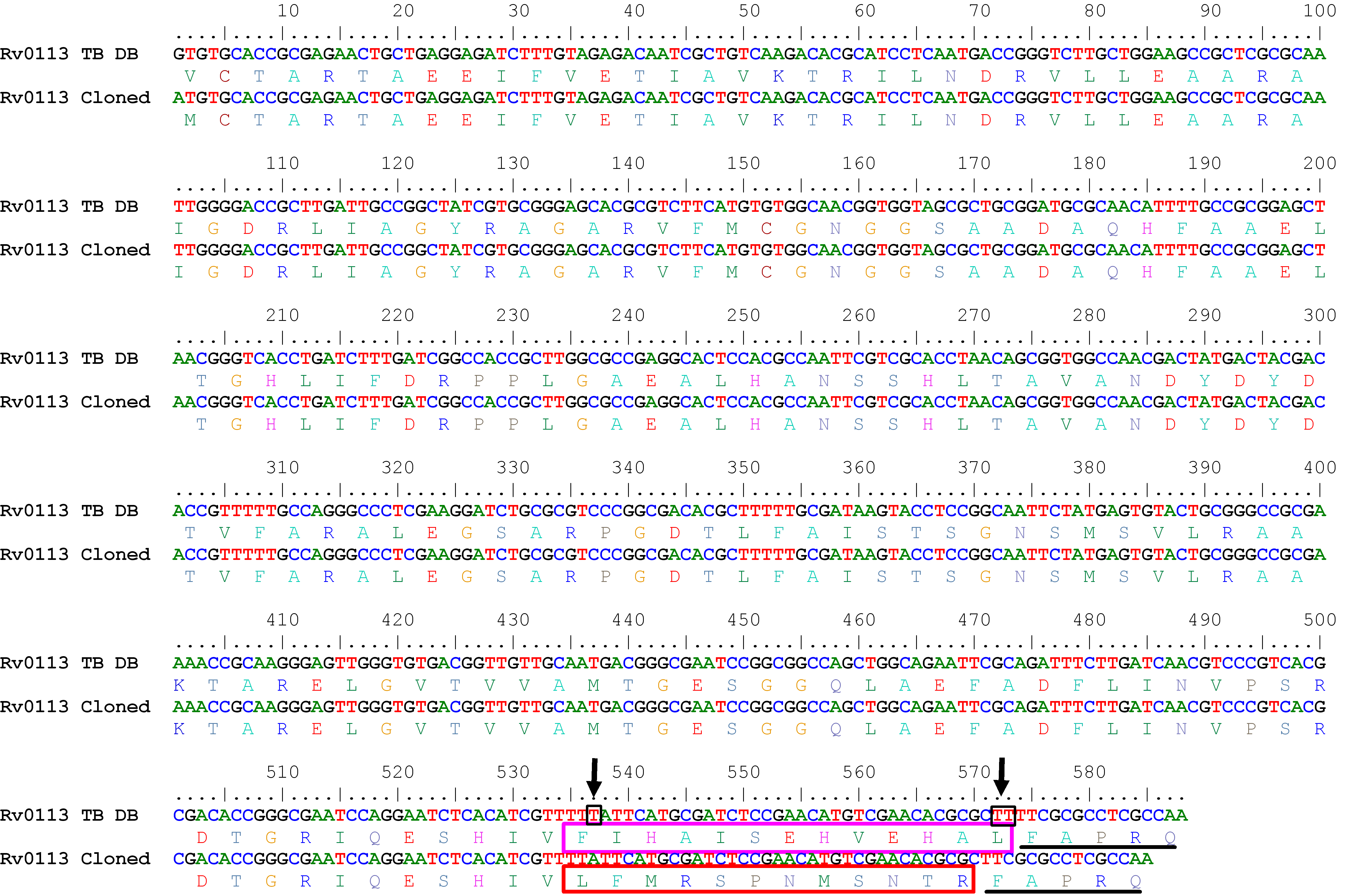

Supplement: Figure S3 — Amino-acid sequence of Rv0113 wild-type Sanger Database reference sequence (Rv0113 TB DB) (http://www.doe-mbi.ucla.edu/TB/) and cloned Rv0113 (Rv0113 Cloned). DNA sequencing of the cloned Rv0113 revealed a single base deletion at bp 537, and a two-base deletion at bp 572 relative to the original Rv0113 reference sequence (black boxes indicated by arrows). This led to the replacement of 13 amino acids near the C-terminus of the original protein (pink box) with a frame-shifted peptide (red box) in the cloned Rv0113. This resulted in a net single amino acid deletion keeping the first and last amino acids in the native frame with no stop codon. (1.06 MB TIF) [file pone.0002387.s003.tif]

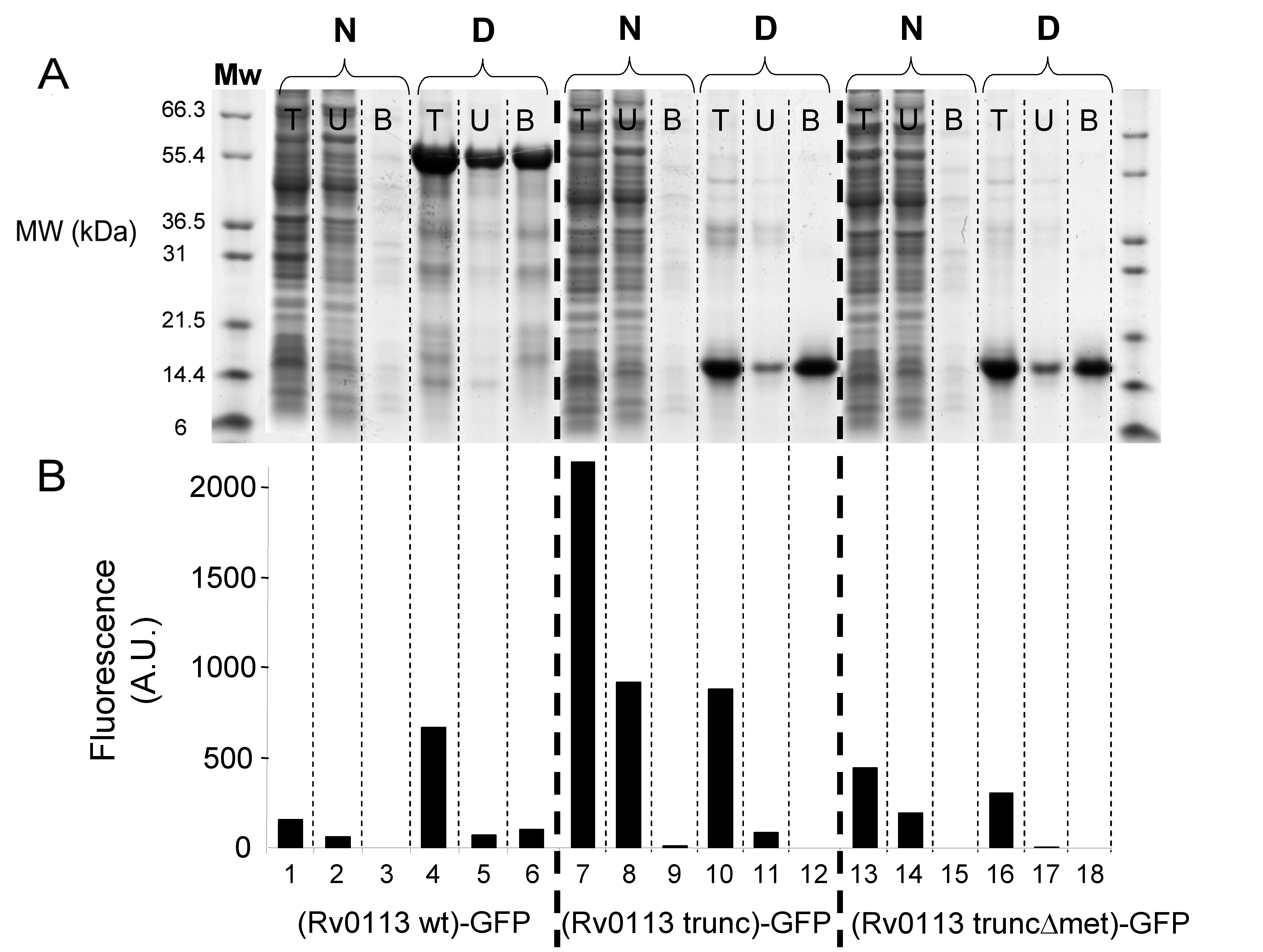

Supplement: Figure S4 — (A) SDS-PAGE of Talon® resin-binding of soluble and insoluble fraction of wild type Rv0113 type ((Rv0113 wt)-GFP), truncated Rv0113 ((Rv0113 trunc)-GFP), and truncated Rv0113 with three methionine-to-leucine substitutions ((Rv0113 truncΔmet)-GFP) variants as N6HIS-X-GFP fusions. Soluble extracts were bound to Talon®beads under native conditions (N), whereas insoluble pellets were unfolded in 9M urea and bound to Talon® resin under denaturing conditions in 9M urea (D). Total extract (T), unbound protein (U) and bound protein (B). (B) Fluorescence of corresponding samples, total extract (T), unbound protein (U) and bound protein (B), measured using a BioTEK plate reader. (2.93 MB DOC) [file pone.0002387.s004.tif]

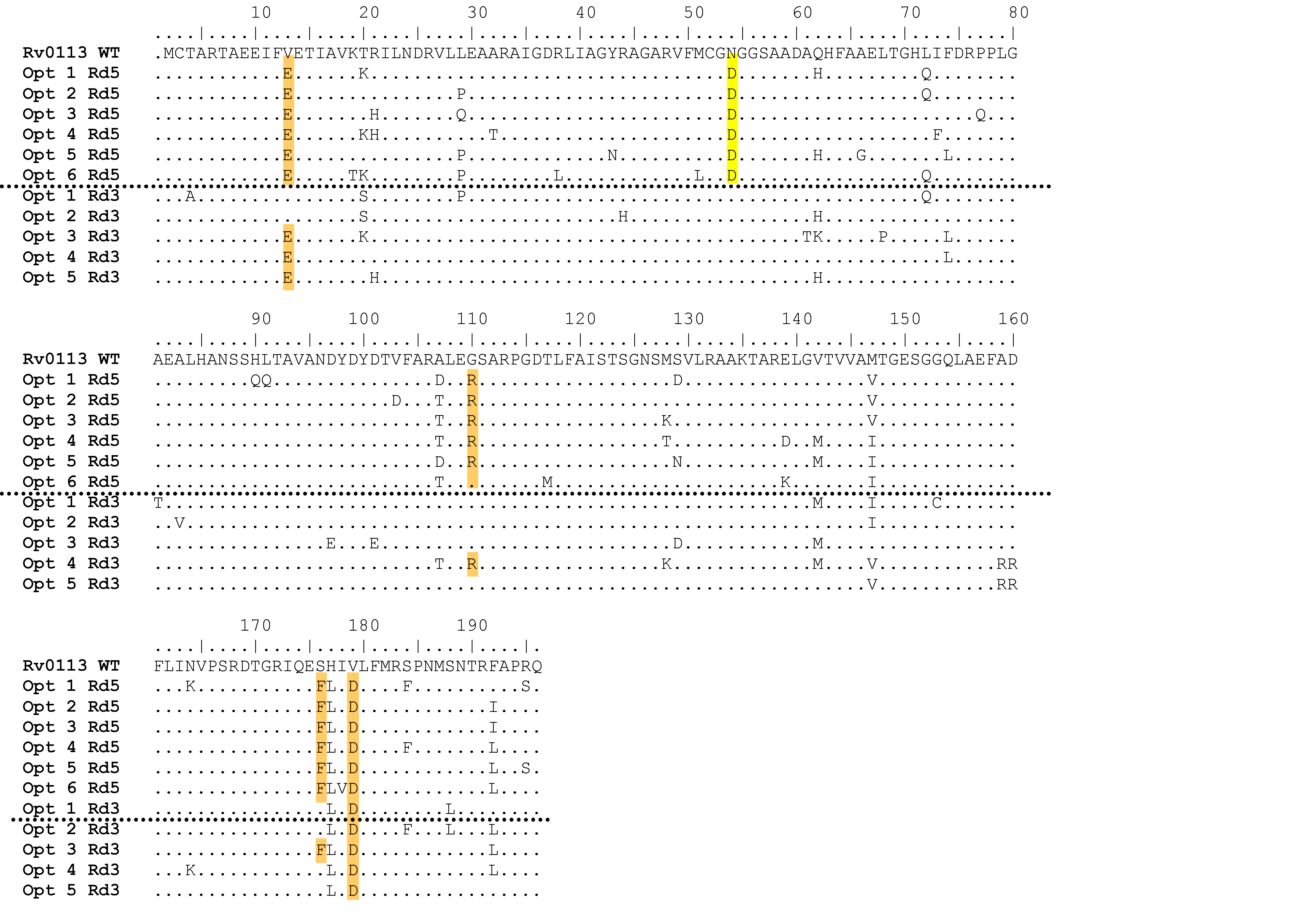

Supplement: Figure S5 — Amino acid sequence alignment of Rv0113 starting variant and brightest mutants from successive rounds of directed evolution using the GFP insertion reporters. DNA sequences of five optima obtained after three rounds (Rd3) in the least stringent FR/SF reporter (shown below the dotted line in each set). Round 5 DNA sequences of six optima obtained after taking the round 3 optima through two additional cycles in the most stringent FR/FR vector (Rd5) (shown above the dotted line in each set). Mutations found in some optima of round 3 were highly enriched after round 5 (orange highlight). One additional mutation N54D appeared only after round 5 and is correlated with increased solubility of the new mutants (yellow highlight). (0.70 MB TIF) [file pone.0002387.s005.tif]
